# Research on the application of remote sensing image super-resolution reconstruction techniques in crop phenology extraction

**DOI:** 10.3389/fpls.2025.1687246

**Published:** 2025-11-05

**Authors:** Hao Han, Ziyi Feng, Yuanji Cai, Wanning Li, Wen Du, Tongyu Xu

**Affiliations:** ^1^ College of Information and Electrical Engineering, Shenyang Agricultural University, Shenyang, China; ^2^ Liaoning Provincial Key Laboratory of Smart Agriculture Technology, Shenyang, China; ^3^ National Digital Agriculture Regional Innovation Center (Northeast), Shenyang, China; ^4^ High-resolution Earth Observation System, Liaoning Forest and Grass Resources and Environment Remote Sensing Research and Application Center, Shenyang, China

**Keywords:** phenology extraction, satellite remote sensing, super-resolution reconstruction, crop phenology, generative adversarial network

## Abstract

Crop phenology is one of the most critical physiological attributes of agricultural crops, serving as a direct indicator of growth status throughout the developmental cycle. With the advancement of phenological research, satellite remote sensing has emerged as a primary monitoring tool due to its large spatial coverage and convenient data acquisition. However, high-resolution remote sensing satellites, which are essential for precise phenological observations, often have long revisit intervals. Additionally, adverse atmospheric conditions such as cloud cover frequently compromise the usability of images on multiple dates. As a result, high-resolution time-series data for crop phenology monitoring are typically sparse, limiting the ability to capture rapid phenological changes during the growing season.To address this challenge, this study focuses on paddy and dryland fields as experimental sites and proposes a novel method for filling temporal gaps in remote sensing data using generative image processing techniques. Specifically, a lightweight super-resolution Generative Adversarial Network (GAN) is developed for image reconstruction. Using the reconstructed dataset, dense time-series monitoring and phenological metric extraction were conducted throughout the crop growing season.(1) The proposed super-resolution reconstruction method achieves structural similarity index (SSIM) and peak signal-to-noise ratio (PSNR) values of 0.834 and 28.69, respectively, outperforming mainstream approaches in reconstructing heterogeneous remote sensing data.(2) Following temporal reconstruction, the revisit intervals of remote sensing imagery for the two test sites improved from 6.40 and 6.63 days to 5.70 and 5.88 days, respectively. To further analyze phenological metrics, four smoothing techniques were applied, among which Savitzky–Golay filtering yielded the most accurate and robust results. Although discrepancies were observed between the results obtained using the reconstructed data and those based on the original datasets, the proposed method demonstrated smaller deviations from benchmark datasets. Compared with conventional interpolation-based gap-filling approaches, the framework demonstrated marked improvements in the accuracy of phenological extraction, while also delivering superior spatial resolution and robustness relative to the Harmonized Landsat and Sentinel (HLS) dataset. Experimental results confirm that the proposed approach effectively fills temporal gaps in satellite imagery, enhances data continuity, accurately captures key phenological turning points, and enables precise crop phenology monitoring at high spatial and temporal resolution.

## 1 Introduction

Phenology refers to the periodic biological events in organisms that have evolved in response to long-term climatic conditions (Schwartz et al., 2002). Vegetation phenology, in particular, serves as a key parameter for characterizing vegetation dynamics on land surfaces. It is one of the most direct indicators of terrestrial ecosystem responses to global climate change (Richardson et al., 2013; Fu et al., 2015; Liu et al., 2022; Li et al., 2023; Xiong et al., 2024; Zhang et al., 2025) modulating numerous feedback pathways between terrestrial ecosystems and the climate system (Wang et al., 2018; Piao et al., 2019; Yue et al., 2023) and influencing nearly all aspects of ecology and biological evolution (Miller-Rushing and Weltzin, 2009; Caparros-Santiago et al., 2021).Over the course of its development, phenological science has established a range of methods for observing and predicting vegetation phenology, which can generally be categorized into three main types: ground-based observations (including manual monitoring, phenocam observations, and flux measurements), remote sensing monitoring, and phenological modeling. Among these, remote sensing has gained widespread application due to its large-scale coverage and efficient data acquisition capabilities (Guan et al., 2014; Mascolo et al., 2016; Steele-Dunne et al., 2017; Wang et al., 2018). However, the extraction of crop phenology remains more challenging, primarily due to the rapid and continuous changes in vegetation indices during crop growth stages, which require high temporal continuity. This challenge has attracted considerable attention in the research community, prompting the development of various time-series gap-filling methods.

Since the early work by Thompson (1980), who used Landsat optical sensors to monitor wheat responses to water stress, optical remote sensing has played a pivotal role in advancing crop phenology research. With the continuous development of remote sensing technology, both spatial and temporal resolution have improved. However, high-resolution imagery remains difficult to obtain consistently, while medium- to low-resolution data are prone to mixed-pixel effects. Moreover, cloud cover and aerosols frequently contaminate satellite-derived observations. Even sensors with daily revisit capabilities, such as MODIS, often yield limited usable data due to these constraints (Zhang et al., 2003; Fraser et al., 2009; Xu et al., 2017).

To address these issues, several scholars have proposed methods for data reconstruction. For instance, Hermance et al. used harmonic models to perform linear fits to missing Landsat data, enabling the prediction of land cover dynamics for arbitrary dates (Hermance, 2007). Although such spatial interpolation techniques are effective in noise reduction and smoothing, they often lack accuracy and real data support for reconstructing missing observations. In the context of multi-sensor time-series reconstruction, Roy et al. utilized heterogeneous data from Landsat-5 and Landsat-7 to generate dense NDVI time series over six agricultural regions in the United States (Kovalskyy and Roy, 2013). Their research group also compared BRDF-corrected reflectance and NDVI values between Sentinel-2A and Landsat-8 under various atmospheric and surface conditions, revealing significant differences when data were not corrected (Zhang et al., 2018). Onojeghuo et al (Onojeghuo et al., 2018). fused MODIS and Landsat data to obtain high spatiotemporal resolution NDVI time series (30 m spatial resolution at 8-day intervals), demonstrating improved accuracy in rice phenology monitoring. Such data fusion approaches have been validated as effective means to enhance crop phenology detection by leveraging the complementary strengths of different satellite systems. Studies employing harmonized Landsat and Sentinel-2 data have also emerged in recent years, though the spatial resolution remains limited to 30 m. Recent research has shown that higher spatial resolution significantly improves phenological detection accuracy (Song et al., 2024). Moreover, high-frequency observations are more suitable for capturing rapid changes in crop growth. However, a single sensor is often insufficient for continuous monitoring, underscoring the need for further exploration of multi-satellite, high-resolution phenological monitoring strategies.With the rapid advancement of generative image processing techniques, deep learning frameworks based on Generative Adversarial Networks have achieved impressive results in remote sensing image tasks. GANs offer powerful capabilities for style transfer and resolution enhancement, enabling cross-sensor resolution reconstruction and spectral harmonization—thereby addressing the key limitations of interpolation and fusion methods that lack data support, as well as discrepancies in resolution and spectral properties among heterogeneous sensors.

In this context, we propose a novel approach that employs a GAN framework to unify spatial resolution and spectral characteristics across heterogeneous satellite imagery. The method generates a time series of remote sensing images at a consistent spatial scale (10 m) and within the same spectral bands, facilitating field-scale, high-resolution crop monitoring and accurate phenological extraction. Our research includes two main innovations: (1) firstly, this study proposes a lightweight generative adversarial network model to train the network and evaluate the quality of the generated images; (2) Secondly, the spatial resolution and spectral range of the annual Landsat-8 image data and the Sentinel-2 image data were unified by using the proposed resolution reconstruction method, and dense time-series data were generated and applied to crop phenological extraction, and the performance of each extraction method was evaluated. The results show that the optical time series data generated by the proposed method has short interval revisit and 10-meter spatial resolution, which significantly improves the accuracy of phenological monitoring, so as to more accurately identify key phenological transition nodes. In addition, this scheme provides a flexible solution to enhance the spatial or temporal resolution of time-series imagery without being limited by the initial data scale, showing great potential for advancing satellite-based phenological research.

## 2 Study area and data sources

### 2.1 Overview of the study area

The study area is located in the southern part of Liaoning Province, near the estuary of the Liao River. The region experiences a temperate, semi-humid monsoon climate, characterized by higher precipitation during spring and summer, and relatively lower rainfall in autumn and winter. The annual average precipitation is approximately 650 mm, with a mean annual temperature of 8.5 °C. The Liaohe Plain, where the study area is situated, is a key protected zone of the northeastern black soil region. It is known as a major agricultural production area in south-central Liaoning, one of China’s most important grain-producing regions (Zhou et al., 2016; Yu et al., 2024).

**Figure 1:**
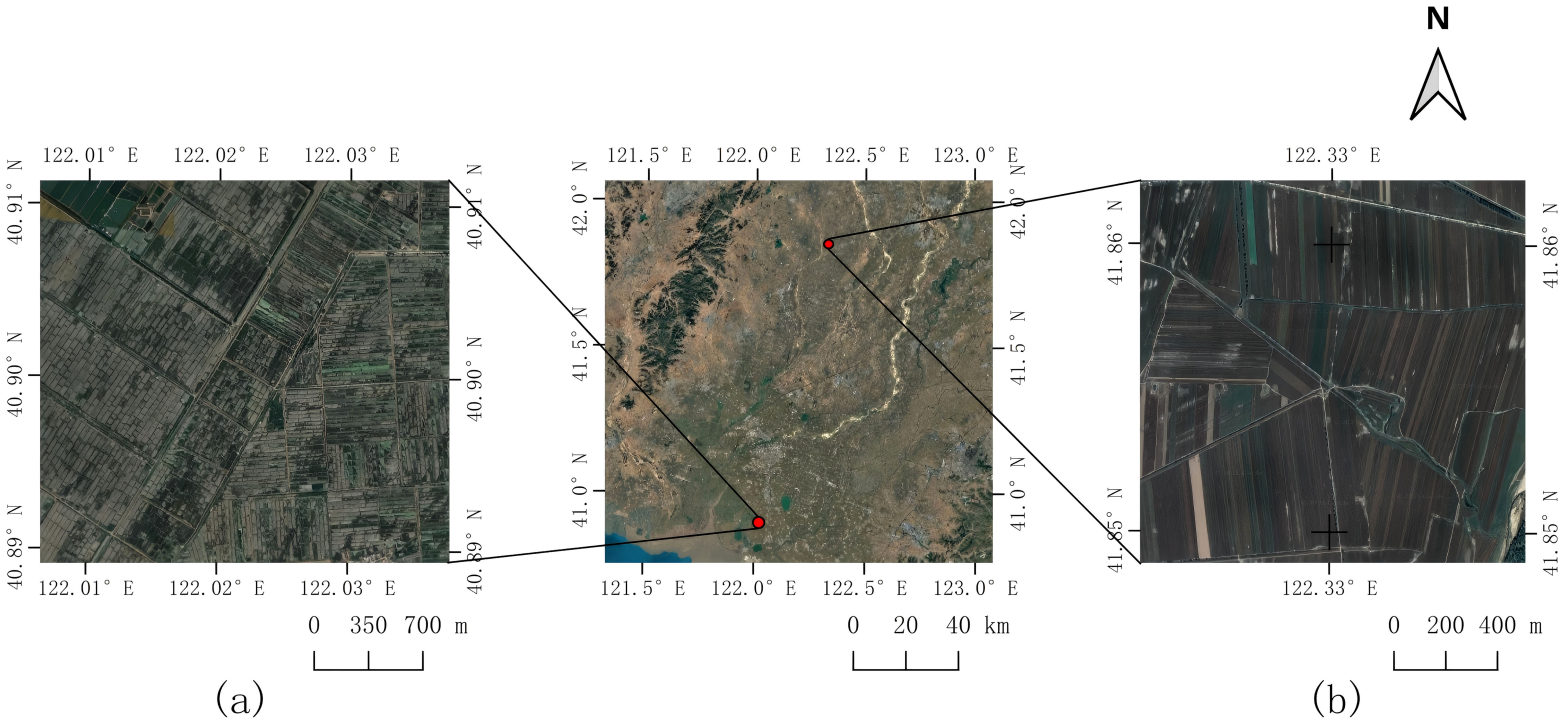
Schematic map of the study area. **(a)** paddy fields, **(b)** dry fields.

For the purposes of this study, extensive tracts of both paddy fields and dryland croplands were selected as experimental sites for phenological analysis. The study area is geographically situated between 40°49′N and 42°05′N latitude and 121°25′E and 123°00′E longitude. The dominant agricultural crops cultivated within this region are rice, maize, and sorghum. A schematic representation of the study area is provided in **Figure 1**.

### 2.2 Data description

#### 2.2.1 Generation of super-resolution remote sensing image data

a) Training Dataset for Super-Resolution Network.

To construct the base dataset for 4× super-resolution reconstruction, we first selected 20 Sentinel-2 images acquired during different seasons across the study area. These images were downsampled to 40 m resolution using bicubic interpolation to simulate low-resolution inputs for large-scale pretraining. Additionally, a set of 79 paired images acquired on the same dates by Sentinel-2 and Landsat-8 was included to form a heterogeneous image dataset, Similarly, these Landsat-8 data are downsampled from 30m to 40m, which is a 4 × resolution difference from Sentinel-2 data, which was used to fine-tune the generative neural network for improved performance on multi-source data.

b) Super-Resolution Image Generation Method.

**Figure 2:**
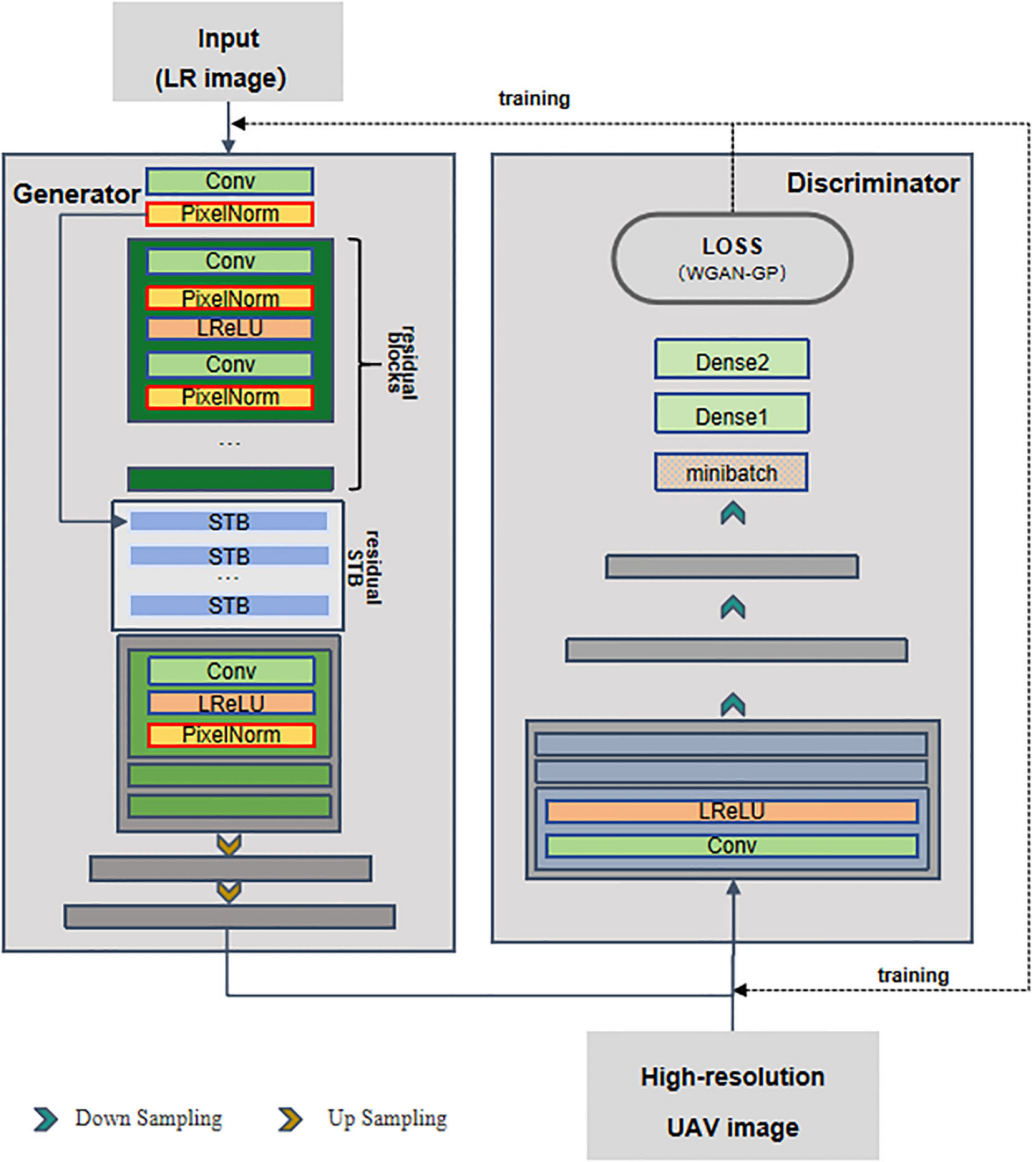
Architecture of the PGT-GAN network.

We designed a compact and lightweight model named PGT-GAN (Progressive Generator Transformer GAN), as illustrated in **Figure 2**, to meet the requirements of super-resolution reconstruction for time-series remote sensing imagery. This architecture balances generation quality comparable to state-of-the-art networks with structural simplicity.The generator consists of two main components: deep feature extraction and resolution enhancement. The low-resolution input images (from step a) are first passed through a series of residual blocks and Swin Transformer modules to extract deep features (Liu, 2021). These features are then progressively upsampled using multiple blocks composed of convolutional layers, pixel normalization layers, and Leaky ReLU (LReLU) activation functions.The discriminator adopts the structure of the PGGAN framework (Karras et al., 2018), performing progressive downsampling through convolutional and LReLU layers. The output is then normalized, and the loss is computed between the generated images and the original high-resolution references to guide network optimization.The network employs the Adam optimizer with a learning rate set to 0.0003, a batch size of 4, and is trained for 200 epochs to achieve high-quality reconstructed images.Regarding the experimental environment, the network was trained on a system equipped with an NVIDIA Quadro P5000 GPU running Ubuntu 20.04, utilizing CUDA version 12.0 and TensorFlow version 2.8.0.

#### 2.2.2 Acquisition of original time-series remote sensing imagery

Sentinel-2 is a series of high-resolution multispectral imaging satellites launched by the European Space Agency (ESA), consisting of two satellites—Sentinel-2A and Sentinel-2B—launched in 2015 and 2017, respectively. Each satellite is equipped with a Multispectral Instrument (MSI), capable of capturing data across 13 spectral bands ranging from the visible and near-infrared to the shortwave infrared regions. The sensor has a swath width of up to 290 km, with spatial resolutions of 10 m, 20 m, and 60 m depending on the spectral band. The orbital parameters of the Sentinel-2 satellites are summarized in **Table 1**.

**Table 1:** Sentinel-2 A/B orbital parameters.

Parameter	Indicator
Orbit type	Sun-synchronous orbit
Orbit height	786km
Orbital inclination	98.5
Regression cycle	10 days

Regarding the acquisition of original time-series imagery, this study collected all cloud-free and noise-free Sentinel-2 images captured in 2023 within the geographic coordinates of 40.84°N–40.92°N and 121.98°E–122.07°E. These data were downloaded from the Copernicus Open Access Hub (https://www.esa.int/) at Level-1C processing. Radiometric and atmospheric corrections were applied using the Sen2Cor plugin developed by the ESA, producing Level-2A data. These corrected images served as the foundational dataset for constructing the annual time series used in continuous monitoring and phenological extraction. In the paddy field area, a total of 57 valid time-series images were obtained, with an average temporal resolution of 6.40 days. For the dryland cropland area, 55 valid images were collected, with an average temporal resolution of 6.64 days. Each image includes the four spectral bands essential for phenological extraction: red, green, blue, and near-infrared (NIR).

### 2.3 Evaluation metrics and comparison methods

#### 2.3.1 Evaluation metrics for super-resolution reconstruction

In the context of single-image super-resolution reconstruction for remote sensing applications, two widely adopted evaluation metrics were selected: the Peak Signal-to-Noise Ratio and the Structural Similarity Index Measure (Hore and Ziou, 2010). PSNR measures the ratio between the maximum attainable power of a signal and the power of distorting noise that compromises the fidelity of its representation. Mathematically, PSNR is defined by Equation 1, where MAX denotes the maximum possible pixel value in the image, and MSE represents the mean squared error.


$$PSNR=10\cdot {\text{log}}_{10}\left(\frac{{\text{MAX}}^{2}}{\text{MSE}}\right)$$

SSIM is another widely adopted metric for assessing image quality, grounded in the hypothesis that the human visual system perceives images by extracting structural information. It serves as a quantitative measure of the similarity between two images. SSIM evaluates image fidelity based on three comparative components: luminance, contrast, and structure, as formulated in Equation 2:


$$SSIM\left(x,y\right)\;=\;\left[l{\left(x,\;y\right)}^{\alpha }\;\cdot \;c{\left(x,\;y\right)}^{\beta }\;\cdot \;s{\left(x,\;y\right)}^{\gamma }\right]$$

Where *l*(x, y) quantifies the luminance difference, c(x, y) measures the contrast difference (represented by the standard deviation of pixel intensities), and s(x, y) evaluates the structural dissimilarity between images.

#### 2.3.2 Comparison and evaluation of phenological metrics

To demonstrate the effectiveness of the proposed method, we compared the phenological metrics extracted using the super-resolution time-series reconstruction approach with those obtained from both interpolation-based gap-filling methods and the original data, under various smoothing conditions. The smoothing models employed in this study have been widely validated for their effectiveness and include Moving Average Smoothing (Moving), Locally Weighted Scatterplot Smoothing (LOWESS) (Cleveland, 1979), Robust LOWESS (RLOWESS) (Wilcox, ), and the Savitzky-Golay (S-G) filter (Chen et al., 2004; Belda et al., 2020). Our objective is to conduct a comprehensive evaluation of the proposed method under different smoothing algorithms and assess its robustness across varying conditions.Among the interpolation techniques commonly used in current phenological studies, Gaussian Process Regression (GPR) (Schulz et al., 2018) is considered one of the preferred methods for filling gaps in time-series imagery. Notably, GPR is also one of the few interpolation approaches capable of providing associated uncertainty estimates. Therefore, GPR was adopted in this study as a benchmark interpolation method to fill remaining gaps in the time series, serving as a comparative reference.

For the accuracy assessment of the extracted phenological metrics, this study employed the HLS dataset as the reference standard. The HLS dataset, released by NASA, is based on atmospherically corrected surface reflectance products from Landsat 8–9 Level-1 and Sentinel-2 Level-1C data. It undergoes multiple preprocessing steps, including atmospheric compensation, view angle correction, and spectral harmonization, to generate a globally consistent time-series product at 30 m spatial resolution. Since its global release in April 2013, HLS data has been widely applied in phenological extraction and validation studies (Claverie et al., 2018; Bolton et al., 2020; Tran et al., 2023).

## 3 Phenology extraction experiment

### 3.1 Experimental workflow diagram

**Figure 3:**
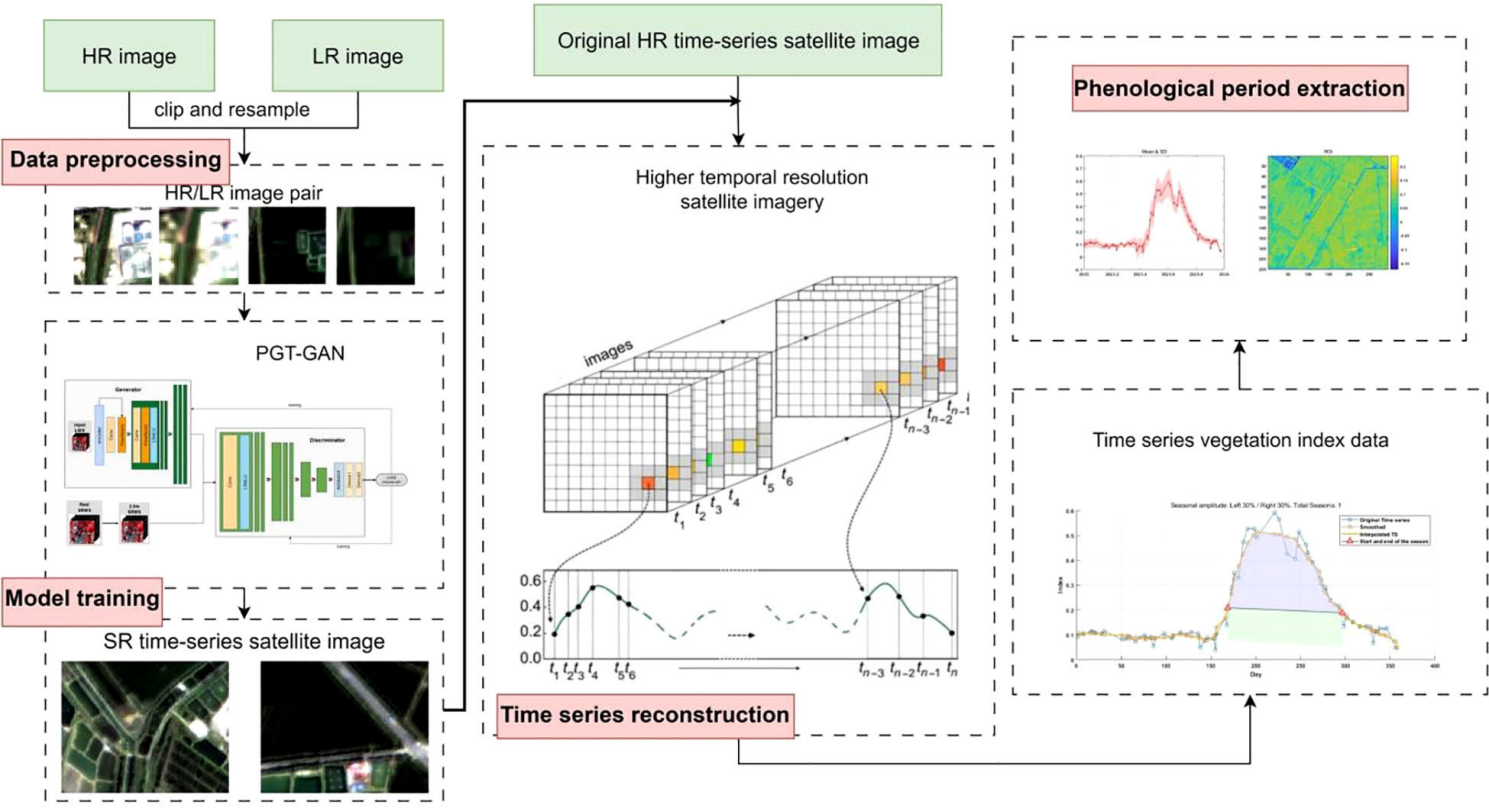
Overall experimental technical roadmap.

The experimental workflow adopted in this study, as schematically illustrated in **Figure 3**, is systematically organized into four main phases: data preprocessing, model training, time-series reconstruction, and phenological parameter extraction. During the data preprocessing stage, input–target pairs required for model training are constructed, while test imagery from heterogeneous satellite platforms is converted into a format compatible with the network architecture. This is specifically implemented by pairing the images and cropping them into corresponding patches of 64×64 and 256×256 pixels, reflecting a four-fold resolution relationship. In the subsequent phase, a generative deep learning model is trained using the prepared dataset, through which Landsat-8 imagery is processed by the generative network and reconstructed into images with a spatial resolution of 10 meters, exhibiting spectral characteristics that closely align with Sentinel-2 observations. The reconstructed images are then integrated chronologically with original Sentinel-2 data to form an enhanced and more densely sampled time series. In the final phenological parameter extraction stage, vegetation indices are computed from the reconstructed imagery, and multiple smoothing methods are applied to the time-series vegetation index data to extract key phenological metrics, including Start of the Season, End of the Season, Length, Max Day, and Amplitude, thereby providing a foundation for comprehensive comparative evaluation and in-depth analytical investigation.

### 3.2 Performance analysis of remote sensing image super-resolution reconstruction

This study validates the resolution reconstruction results of the proposed method using heterogeneous image pairs consisting of Sentinel-2 (10m resolution) and Landsat-8 (bicubic interpolated to 40m resolution) data acquired on November 4, 2023. Quantitative comparisons presented in **Table 2** demonstrate that the proposed super-resolution reconstruction network exhibits marginal superiority over current state-of-the-art super-resolution networks.

**Table 2:** Quantitative comparison of super-resolution reconstruction results.

Method	SSIM	PSNR	RMSE
ESRGAN	0.819	27.17	0.377
pix2pix	0.813	27.5	0.36
SwinIR	0.83	28.56	0.363
PGT-GAN	0.834 ± 0.002	28.69 ± 0.05	0.355 ± 0.03

**Figure 4:**
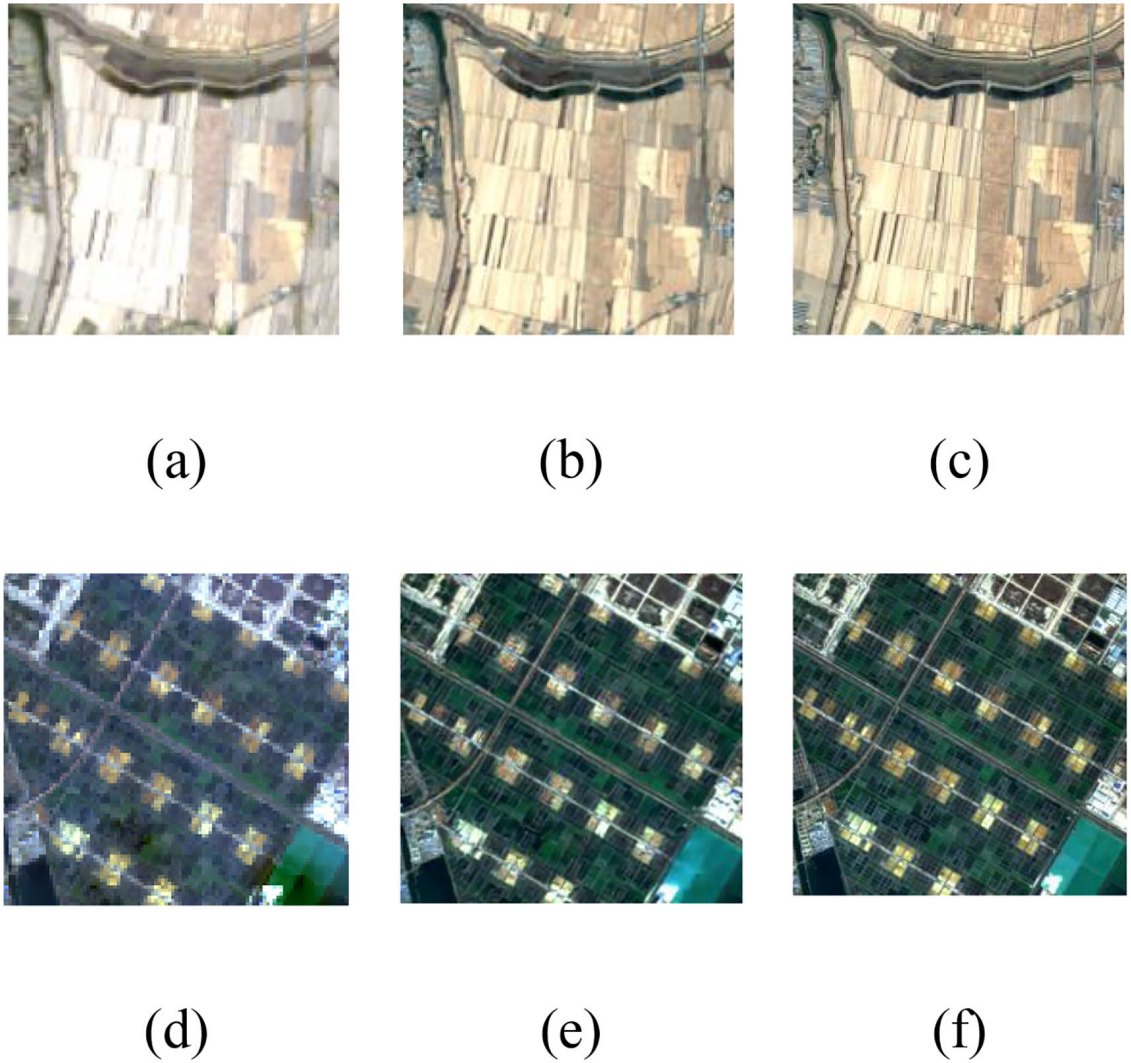
Comparison of results of super-resolution reconstruction methods. **(a)**, **(d)** Landsat-8 images, **(b)**, **(e)** reconstruction results of the proposed method, **(c)**, **(f)** Sentinel-2 images.

**Figure 4** presents the detailed reconstruction results of the proposed method. The results demonstrate that our approach exhibits robust image restoration capability, generating reconstructed images with rich textural details and minimal chromatic aberration compared to the reference images.

**Figure 5:**
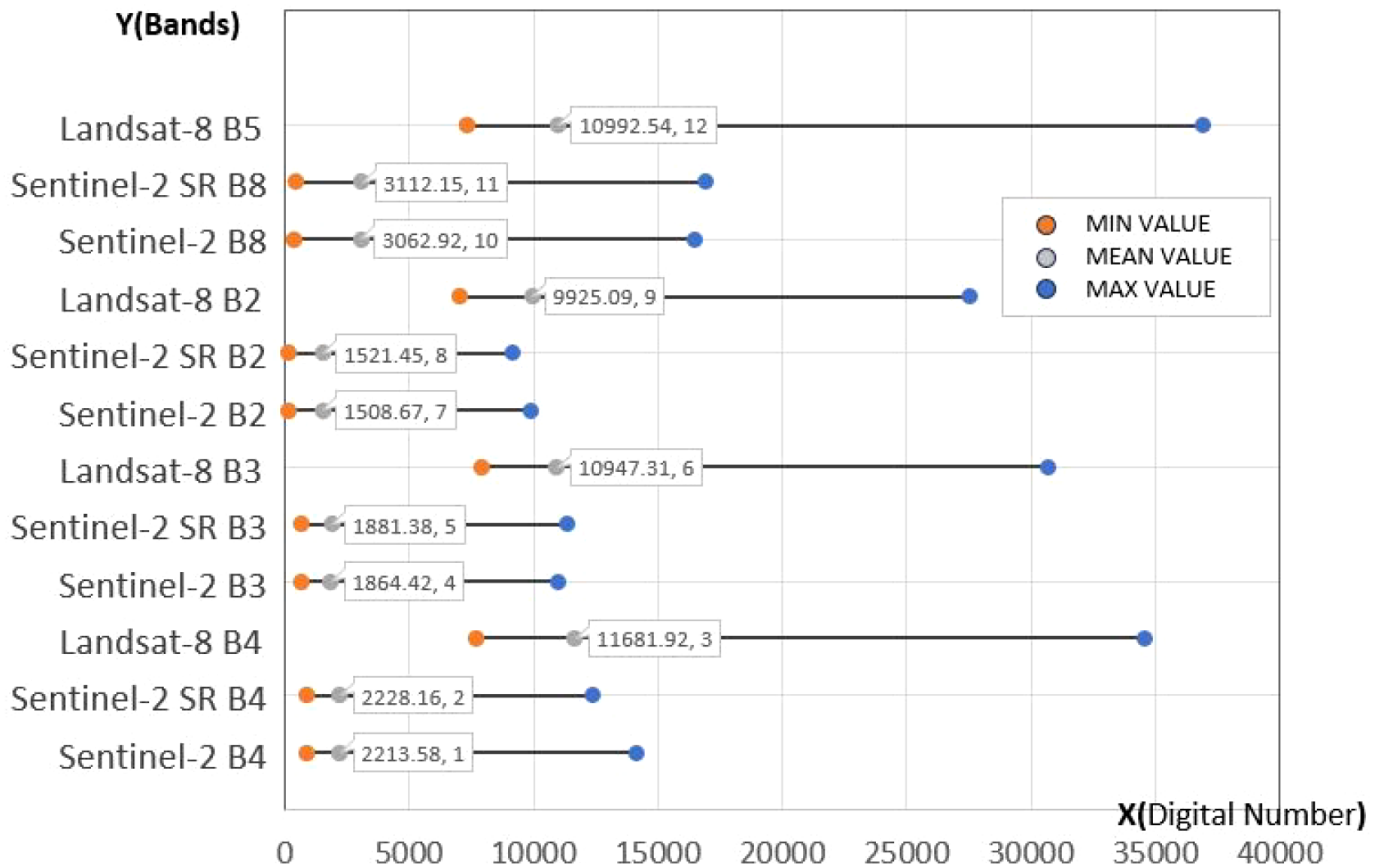
Comparison of the resolution reconstruction results across spectral bands with the original Landsat-8 and Sentinel-2 images.

To better evaluate the reconstruction performance across different spectral bands and assess the spectral discrepancies between the reconstructed and original data, we statistically analyzed the maximum, minimum, and mean digital number (DN) values for each band, as shown in **Figure 5**. The comparison reveals substantial differences in DN values between Landsat-8 and Sentinel-2 across all bands. However, after applying GAN-based super-resolution reconstruction and spectral transfer, the reconstructed images exhibit pixel values highly similar to those of the original Sentinel-2 images, with mean DN values closely matching the originals. This indicates that the reconstructed images maintain a high degree of spectral fidelity to the original data, supporting their suitability for subsequent heterogeneous remote sensing phenological extraction.

### 3.3 Phenology extraction experiment and results

**Figure 6:**
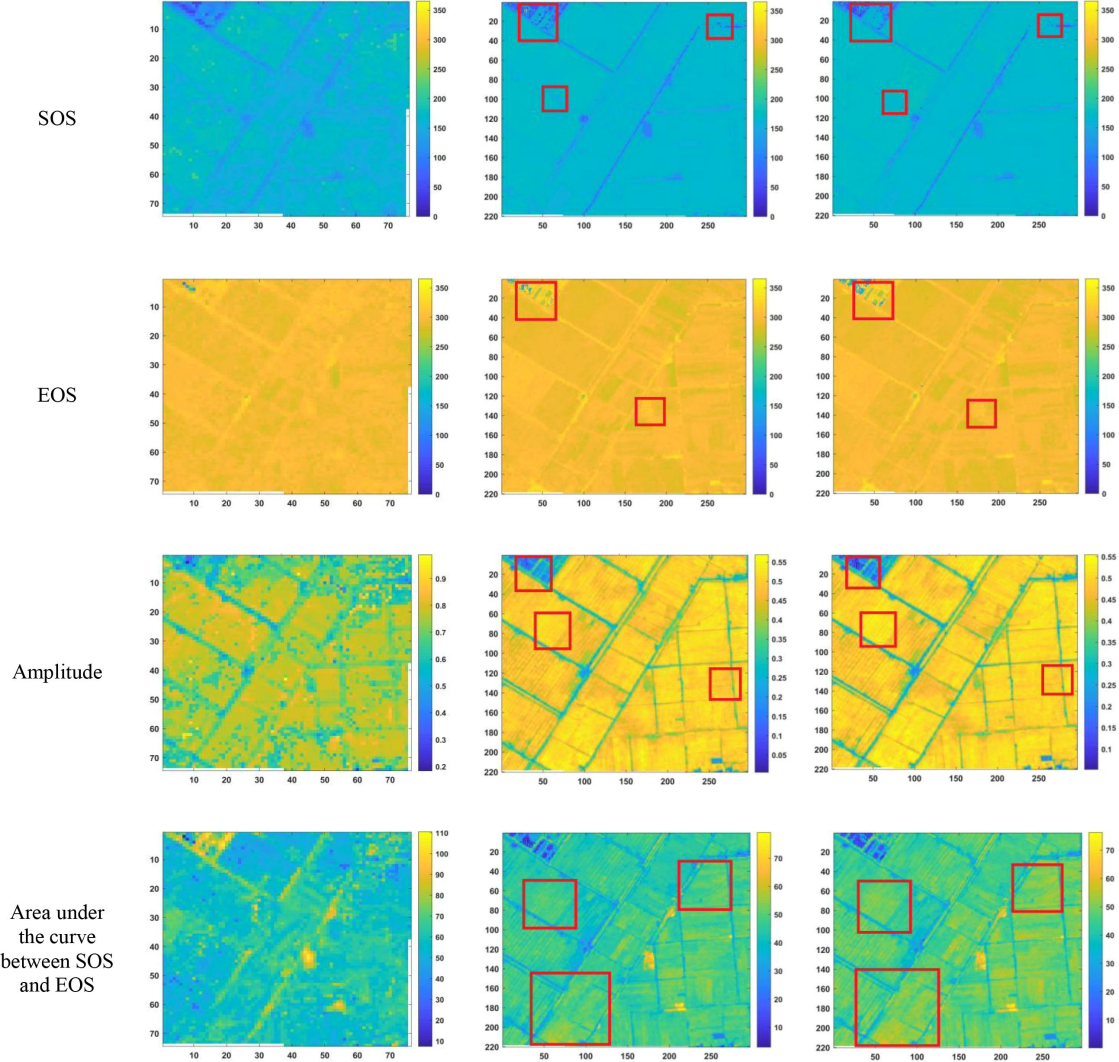
Phenological indicators extracted by the Moving Smoothing method (from left to right, the results extracted from HLS-30m data, the results extracted from Sentinel-2 raw data, and the results extracted after temporal reconstruction using this method).

**Figure 7:**
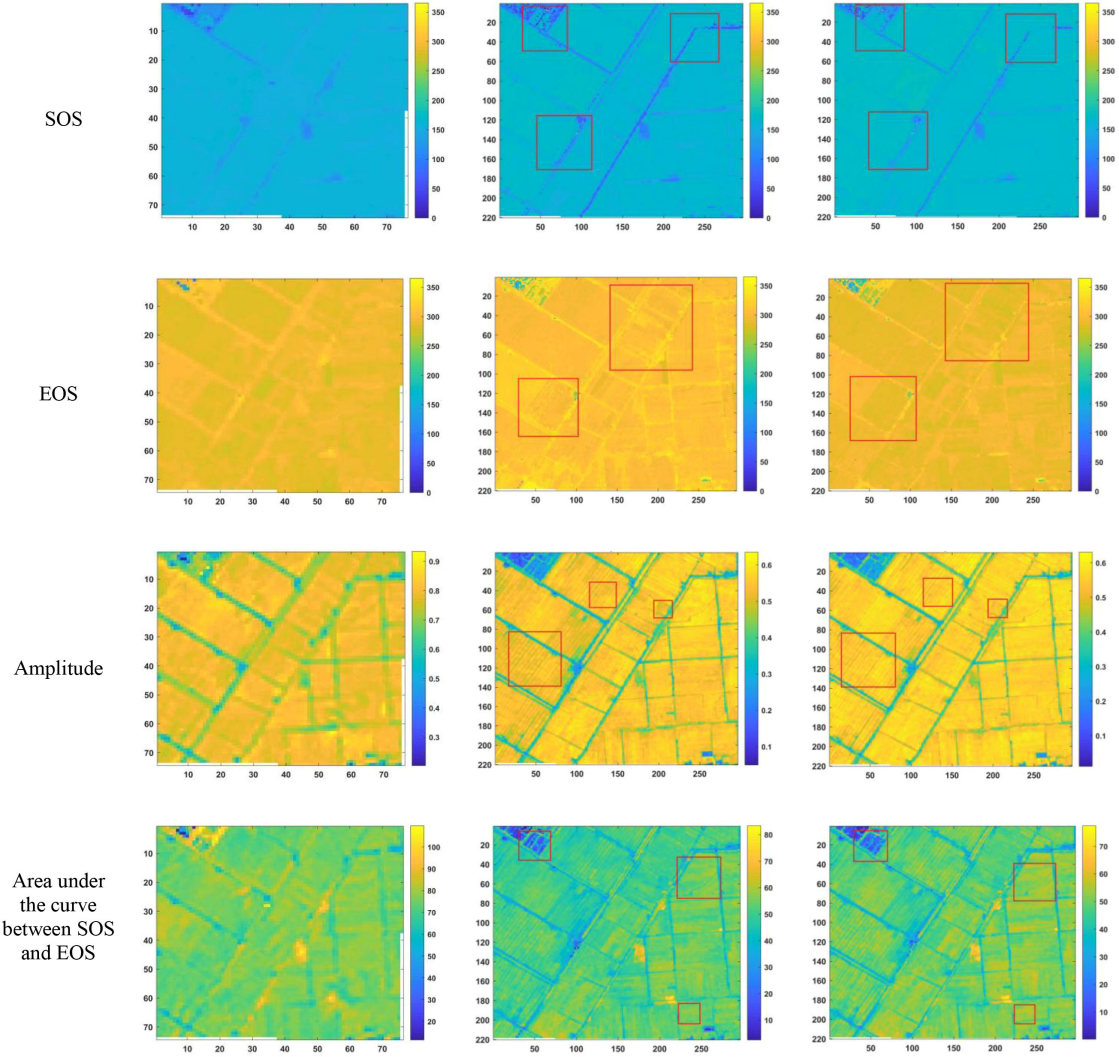
Phenological indicators extracted by the Savitzky-Golay filter method (from left to right: results from HLS-30m data extraction, results from Sentinel-2 raw data extraction, and results after temporal reconstruction using the method in this paper.)

**Figure 8:**
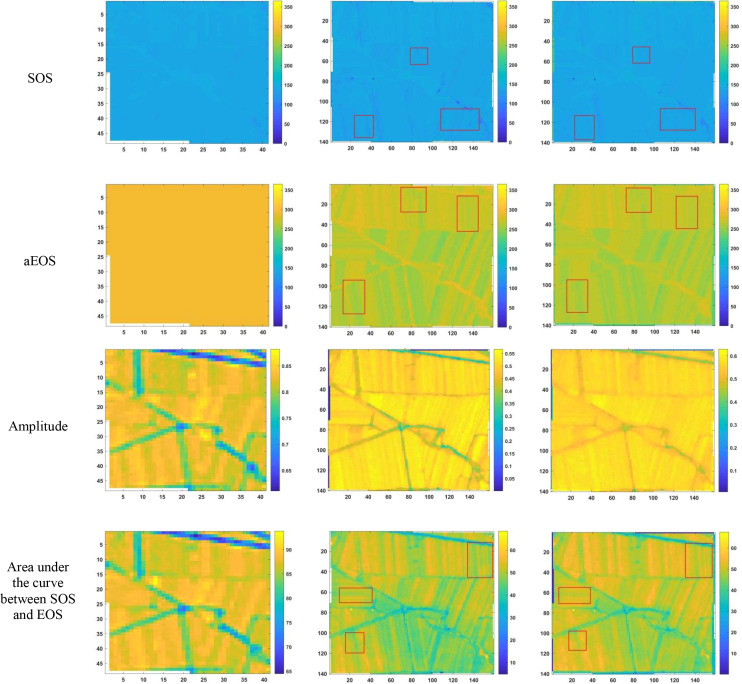
Phenological indicators of dry land extracted using the LOESS filtering method (from left to right are the extraction results from HLS-30m data, the extraction results from raw Sentinel-2 data, and the extraction results after time series reconstruction using the method proposed in this paper).

**Figure 9:**
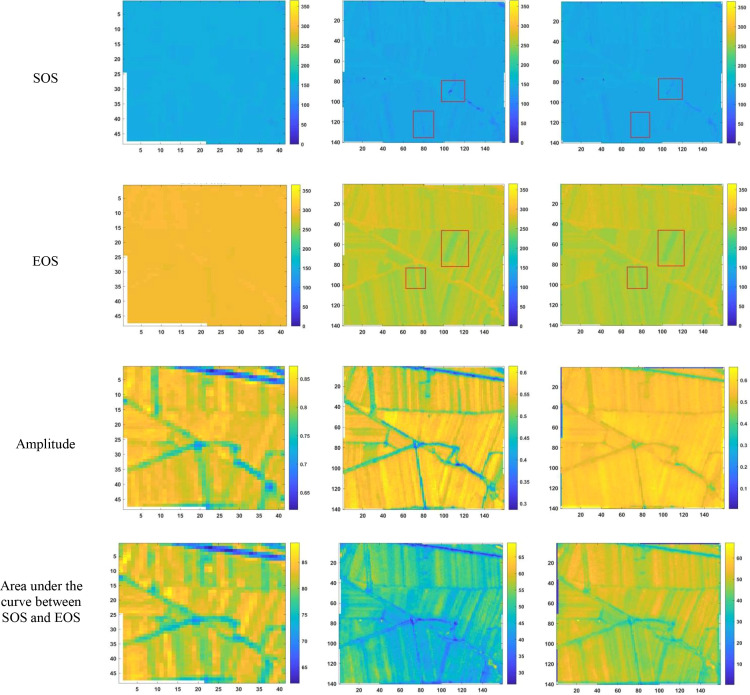
Phenological indicators of dry land extracted using the Savitzky-Golay filtering method (from left to right: results extracted from HLS-30m data, results extracted from raw Sentinel-2 data, and results extracted after temporal reconstruction using the method described in this article).

After reconstructing the images and filling gaps in the satellite time-series data, four different smoothing methods were applied to extract annual phenological metrics for paddy fields and drylands using the NDVI index. These metrics included the start of season (SOS), end of season (EOS), amplitude (the difference between the maximum and mean values around seasonal minima), seasonal integral (area under the curve between SOS and EOS), and Length of Season (difference between SOS and EOS), in order to evaluate the practical effectiveness of the proposed method. **Figures 6**, **7** present the extraction results for paddy fields using the Moving Average smoothing method and Savitzky-Golay filter, respectively. **Figures 8**, **9** show the results for drylands using the LOESS filter and Savitzky-Golay filter, respectively.

**Figure 10:**
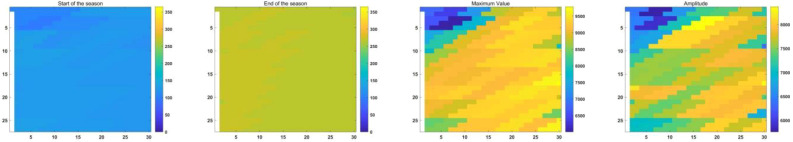
Schematic diagram of various phenological indicators extracted from MODIS data.

As shown in **Figures 6**-**9**, under the same smoothing methods, the reconstructed time-series data exhibit significant differences from the original data in the mapping of various phenological metrics. SOS and EOS extracted using the Moving Average and Savitzky-Golay filtering methods reveal multiple discrepancies. The largest difference is observed in the mapping of maximum date extracted by the Moving Average method, where the maximum dates from the original data tend to be later. The reconstructed images substantially reduce the occurrence of outlier points when extracting SOS and EOS. In experiments using Savitzky-Golay filtering, the extracted EOS dates are generally earlier, with visually noticeable differences in the maximum date and amplitude.High spatiotemporal resolution data demonstrate better robustness, most clearly reflected in the Moving Average method results shown in **Figure 6**. The HLS dataset exhibits considerable noise in phenological metric extraction at the field scale, whereas such noise is absent in the reconstructed data. Across all fields and methods, phenological results extracted from both the original Sentinel-2 images and the reconstructed images are more detailed compared to those from HLS and MODIS data. Although MODIS offers high temporal resolution, as illustrated in **Figure 10**, it is only suitable for large-scale, broad-area detection. The 10 m resolution reconstructed data capture detailed phenological variations between fields more effectively than the 30 m resolution harmonized HLS data, which is particularly critical for precise monitoring in agricultural areas and further highlights the necessity of high-resolution phenology extraction.

**Table 3** and **Table 4** presents the average SOS, EOS, Length of Season, date of maximum value, maximum value, and amplitude extracted by each method for the study area.

**Table 3:** Comparison of the extraction results of phenological indicators in paddy field after image reconstruction.

Method	Data	SOS	EOS	Length	Max day	Amp
Moving	Original	169	296	128	221	0.455
	Sentinel-2	(17-06-23)	(23-10-23)		(09-08-23)	
	After SR	168	294	126	201	0.449
	reconstruction	(16-06-23)	(20-10-23)		(20-07-23)	
RLOWESS	Original	173	295	122	221	0.522
	Sentinel-2	(21-06-23)	(21-10-23)		(09-08-23)	
	After SR	169	294	124	201	0.532
	reconstruction	(18-06-23)	(20-10-23)		(20-07-23)	
LOWESS	Original	170	293	124	226	0.476
	Sentinel-2	(18-06-23)	(20-10-23)		(14-08-23)	
	After SR	171	293	123	221	0.484
	reconstruction	(19-06-23)	(20-10-23)		(09-08-23)	
GPR	Original	160	299	140	221	0.453
	Sentinel-2	(08-06-23)	(26-10-23)		(09-08-23)	
	After SR	161	299	139	221	0.409
	reconstruction	(09-06-23)	(26-10-23)		(09-08-23)	
Savitzky Golay	Original	167	293	126	221	0.455
	Sentinel-2	(15-06-23)	(20-10-23)		(09-08-23)	
	After SR	170	293	123	221	0.499
	reconstruction	(18-06-23)	(20-10-23)		(09-08-23)	
	HLS	172	294	122	218	0.603
		(20-06-23)	(21-10-23)		(16-08-23)	

**Table 4:** Comparison of the extraction results of phenological indicators in dry field after image reconstruction.

Method	Data	SOS	EOS	Length	Max day	Amp
Moving	Original	138	266	128	171	0.485
	Sentinel-2	(05-06-23)	(07-10-23)		(05-07-23)	
	After SR	140	265	126	176	0.5
	reconstruction	(04-06-23)	(07-10-23)		(10-07-23)	
RLOWESS	Original	140	275	135	171	0.516
	Sentinel-2	(04-06-23)	(17-10-23)		(05-07-23)	
	After SR	141	269	129	176	0.533
	reconstruction	(05-06-23)	(11-10-23)		(10-07-23)	
LOWESS	Original	141	269	128	171	0.531
	Sentinel-2	(05-06-23)	(11-10-23)		(05-07-23)	
	After SR	141	266	125	186	0.535
	reconstruction	(05-06-23)	(08-10-23)		(20-07-23)	
GPR	Original	125	273	148	191	0.444
	Sentinel-2	(20-05-23)	(15-10-23)		(25-07-23)	
	After SR	124	274	150	191	0.438
	reconstruction	(19-05-23)	(16-10-24)		(25-07-23)	
Savitzky Golay	Original	141	263	122	176	0.595
	Sentinel-2	(05-06-23)	(05-10-23)		(10-07-23)	
	After SR	141	269	128	183	0.68
	reconstruction	(05-06-23)	(11-10-23)		(17-07-23)	
	HLS	146	273	126	205	0.758
		(10-06-23)	(15-10-23)		(08-08-23)	

These calculations represent the mean values per pixel, even minor numerical differences imply substantial variations across the entire map. Quantitative comparisons reveal that, relative to interpolation-based gap-filling methods, our proposed filling approach consistently shows some degree of difference from the original results across all smoothing methods.

The differences in SOS and EOS fall within ±4 days and ±2 days, respectively, while the Length of Season differs within ±2 days.This study uses phenological metrics extracted from the HLS 30 m dataset as the benchmark for accuracy evaluation. Compared to the original data, the results extracted by our method show smaller date discrepancies relative to HLS 30 m. For both experimental sites, the differences in SOS and EOS extraction are within ±5 days.

## 4 Discussion

The accurate extraction of crop phenological stages represents a critical prerequisite for a wide range of downstream applications in satellite-based remote sensing (Rizos, 2025; Ruan et al., 2023). The present analysis reveals that persistent temporal gaps in image time series substantially compromise the reliability of phenological parameter retrieval, with this effect being particularly pronounced in high-resolution temporal datasets. Annual surface reflectance time series are frequently characterized by extensive, consecutive, and recurrent data voids during key crop growth phases, predominantly attributable to persistent cloud cover and precipitation events in estuarine regions. Such data gaps considerably complicate the derivation of phenological metrics. Specifically SOS and EOS parameters from original imagery demonstrate substantial temporal discontinuities, while the corresponding time-series profiles exhibit marked instability. Although datasets such as MODIS and Harmonized Landsat Sentinel datasets provide enhanced temporal resolution compared to single-sensor products, they remain insufficient to meet the exacting demands of precision phenological monitoring applications.The application of super-resolution reconstruction techniques has demonstrated considerable efficacy in addressing these limitations. Our methodology successfully compensates for missing high-resolution observations during crucial phenological stages, thereby substantially improving the temporal continuity and quantitative accuracy of derived crop growth parameters. This advancement enables more robust monitoring of agricultural phenology than conventional approaches reliant solely on original sensor data.

**Figure 11:**
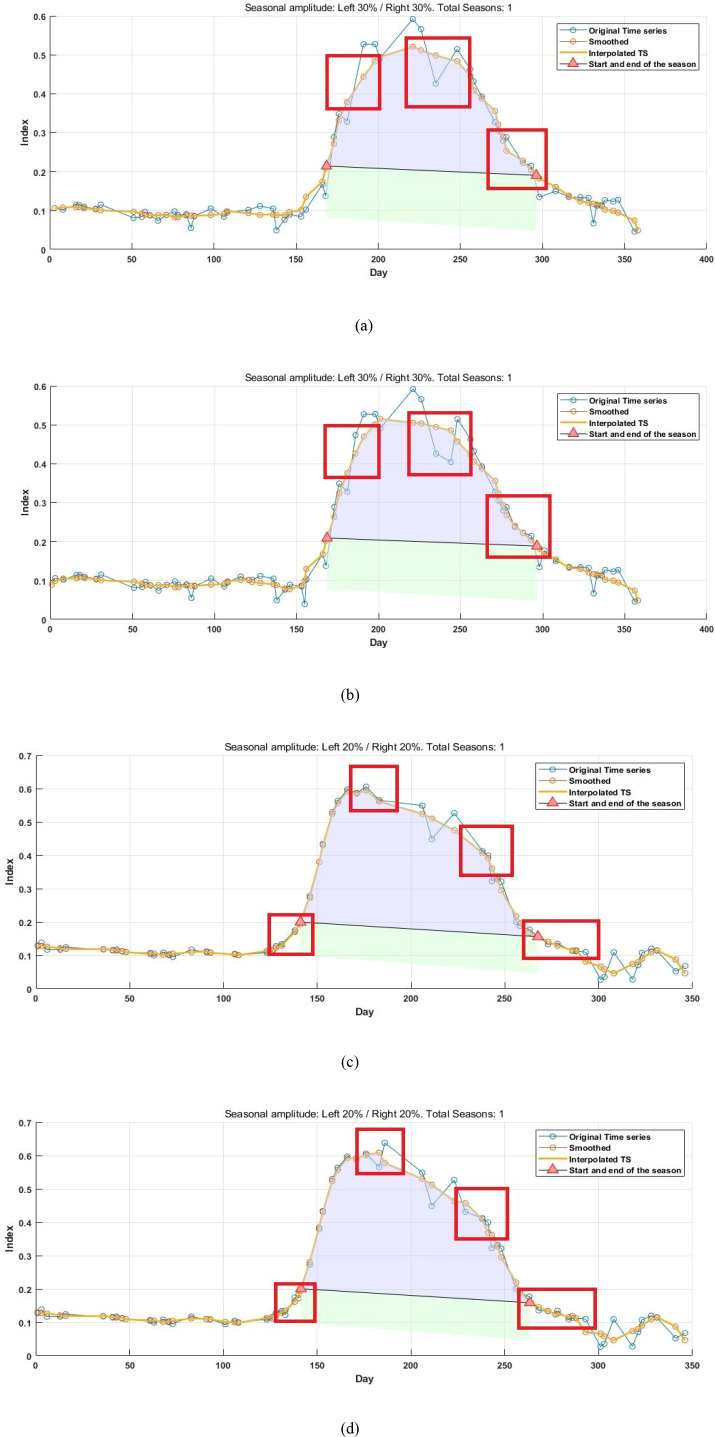
Comparison of the time series curves of original data and reconstructed data **(a)** Original data in paddy field area **(b)** Reconstructed data in paddy field area using the method in this paper **(c)** Original data in dry field area **(d)** Reconstructed data in dry field area using the method in this paper.

**Figure 11** depicts the reconstructed time-series values alongside the phenological indicators extracted using the S-G smoothing method, which demonstrated the greatest robustness among the tested approaches. As illustrated by the time-series curves in **Figures 11b, d** the proposed reconstruction method effectively generates valid vegetation index data within the study area. important for mitigating the lack of high-resolution satellite imagery during key stages of crop development, which otherwise constrains the accurate detection of phenological transitions. Relative to the original time series, the reconstructed data exhibits greater density, with the number of images per year for paddy fields and drylands increasing from 57 and 55 to 64 and 62, respectively, and the average revisit intervals reduced from 6.40 days and 6.63 days to 5.70 days and 5.88 days. At critical phenological transition points (highlighted in red in the figures), the reconstruction method successfully fills data gaps caused by cloud cover, noise, and other factors, resulting in peak shifts, slope increases, and changes in phenological dates. These findings align with the results shown in **Figures 6**-**9**. Future work could extend this analysis to regions characterized by more severe temporal data voids during critical growth stages to further evaluate the applicability of the method for phenological monitoring. It should also be noted that this study primarily verifies the feasibility of single-image super-resolution reconstruction for satellite image time-series reconstruction and phenological extraction, while comparing the outputs against those derived from the original imagery, the HLS 30-m dataset, and MODIS products. The HLS 30-m dataset serves as a reference for accuracy validation of the reconstructed imagery. Nevertheless, more rigorous validation would require integration with ground-based observations, such as PhenoCam data. In future research, we will further verify the reliability of the reconstructed images and derived phenological metrics using ground-truth data.

## 5 Conclusion

Phenology extraction technology plays a critical role in agricultural monitoring and decision support systems. High spatiotemporal resolution data enable precise identification of key growth stages—such as emergence, heading, and maturation—while facilitating early detection of crop developmental anomalies and timely warnings of environmental stressors like drought and frost. Moreover, this technology provides essential data support for subsequent studies on carbon cycle dynamics and climate change.This study introduces a novel framework for phenological extraction that leverages generative remote sensing image reconstruction techniques. The proposed approach is built upon a GAN architecture, designed to simultaneously perform resolution enhancement and spectral transformation of heterogeneous imagery. effectively generating synthetic data to fill temporal gaps in medium-resolution Sentinel-2 time series. A lightweight PGT-GAN model was developed to extract multiple deep features, thereby capturing both spectral and spatial characteristics throughout the resolution reconstruction process. Quantitative evaluation demonstrates the model’s superior performance in both SSIM and PSNR metrics, highlighting its capacity to reconstruct fine-scale textures and spatial details in regions of missing data while preserving high fidelity to the original imagery.Additionally, evaluations of spectral transfer consistency across multiple bands confirm that the method largely maintains spectral integrity throughout the reconstruction process. Importantly, this framework enables high-accuracy resolution reconstruction across an entire annual cycle within a single training process and, unlike conventional spatiotemporal fusion approaches, does not require the availability of precisely co-registered high-resolution reference images. Experiments on real Sentinel-2 and Landsat-8 datasets were conducted using the reconstructed time series, with results compared against phenology extraction from the original data, GPR interpolation, HLS 30-m, and MODIS datasets. The findings demonstrate robust performance across all comparison groups, and validation against the HLS-30m phenological indicators confirms the accuracy of our extraction results.

By integrating remote sensing image super-resolution with time series reconstruction, the proposed framework provides a practical and effective solution for generating high-quality surface observations. The resultant denser reconstructed time series enables precise detection of crop phenological transitions and supports phenology extraction at enhanced spatiotemporal resolutions, thereby advancing the capabilities for land surface phenology monitoring. In practical applications, the utilization of super-resolution reconstructed data with high spatiotemporal resolution facilitates the establishment of quantitative relationships between phenological stages and yield formation, thereby significantly enhancing the accuracy of yield prediction. Furthermore, phenological information provides a scientific basis for optimizing agricultural practices—for instance, by enabling variable-rate fertilization strategies tailored to specific growth phases such as the tillering stage, or by triggering preventive measures during frost-sensitive periods. The integration of these applications further supports intelligent, data-driven management across the entire agricultural production chain. In future research, we plan to validate the proposed method using ground-based datasets and multi-source remote sensing data such as PlanetScope and the Gaofen series.

## Data Availability

The raw data supporting the conclusions of this article will be made available by the authors, without undue reservation.
